# Microvascular Changes Are Associated with Proteinuria and EMG Changes in Patients with Type 2 Diabetes Using Video Capillaroscopy

**DOI:** 10.3400/avd.oa.23-00066

**Published:** 2023-12-22

**Authors:** Alireza Rajaei, Pooneh Dehghan, Nikoo Emtiazi, Azadeh Afzalnia, Faraneh Farsad, Seyed Mohamad Hosseinian

**Affiliations:** 1 Rheumatology Department, Loghman Hakim Hospital, Shahid Beheshti University of Medical Sciences, Tehran, Iran; 2 Radiology Department, Taleghani Hospital, Shahid Beheshti University of Medical Sciences, Tehran, Iran; 3 Department of Pathology, Firoozgar Hospital, Iran University of Medical Sciences, Tehran, Iran; 4 Rajaei Cardiovascular, Medical and Research Center, Iran University of Medical Sciences, Tehran, Iran; 5 Rheumatology Department, Shafa Hospital, Kerman University of Medical Sciences, Kerman, Iran

**Keywords:** diabetes, capillaroscopy, microvascular changes, proteinuria, EMG changes

## Abstract

**Objectives:** Video capillaroscopy is a diagnostic method for evaluating microvascular changes in type 2 diabetes mellitus (T2DM). This study evaluated microvascular changes, including microvascular architecture, capillary distribution (morphology and density), and angiogenesis conditions in T2DM patients via video capillaroscopy.

**Methods:** A total of 256 patients with T2DM enrolled in this study. Based on electromyography (EMG)–nerve conduction velocity results, patients were divided into patients with normal and abnormal EMG. Microalbuminuria was assessed using biochemical urine analysis. Finally, video capillaroscopy was performed to evaluate changes in microvascular architecture, capillary distribution, and angiogenesis status.

**Results:** The differences between microalbuminuria in patients with normal and abnormal EMG were not significant. Other microvascular changes were not significant between normal and abnormal EMG groups. The patients with greater microalbuminuria were at risk of abnormal EMG 2.8 times higher than those with fewer microalbuminuria (odds ratio = 2.804; 1.034–7.601). However, EMG is not a risk factor for microvascular architecture alternation in T2DM (odds ratio = 1.069; 0.323–3.546).

**Conclusions:** Microvascular alternations are common in T2DM and early detection of these changes could help to avoid the progress of nephropathic complications. Also, video capillaroscopy provides a promising diagnostic method for the detection of microvascular alternations in T2DM.

## Introduction

Type 2 diabetes mellitus (T2DM) has been known as a global public health threat with increasing prevalence worldwide. In 2014, the International Diabetes Federation reported that at least 387 million people were living with diabetes mellitus (DM) and predicted that this number will climb to almost 600 million people in 2035.^1^^–^^3)^

T2DM is a form of hyperglycemia and can cause many complications, including vascular complications, nephropathy, and retinopathy.^4)^ About half of patients with diabetes demonstrate signs of kidney damage during their lifetime. Diabetic nephropathy is the most common kidney complication in DM.^5)^ Initially, patients with diabetes have a natural secretion of albumin in urine called early microalbuminuria. The rate of microalbuminuria increases gradually but some studies stated that there is no albuminuria in half of patients with diabetes with renal failure.^6)^ Therefore, it seems necessary to use other diagnostic approaches to detect nephropathy in diabetic patients.

One of these diagnostic methods is video capillaroscopy, a noninvasive, easy, and safe diagnostic method for evaluating microcirculation of small vessels in different diseases, such as DM. The high magnification provided by video capillaroscopy allows more detailed investigations on capillary architecture. Also, video capillaroscopy helps evaluate capillary density, capillary morphological abnormalities, and microvascular architecture.^7^^,^^8)^ As we know, changes in the microvasculature are considered to play an important role in the pathogenesis of metabolic disease. Microangiopathy is an important complication of both types of diabetes. Involvement of small vessels initially occurs in the kidney and retinal vessels. Hyperglycemia results in glycosylation of structural proteins in the small arterial wall, including capillaries, arterioles, and venules. After this improper glycosylation, macrophage receptors detect and destroy vessels’ proteins, leading to microangiopathy. It is assumed that capillaroscopy could be a potentially useful tool for detecting microangiopathic complications of DM and reducing development of nephropathic abnormalities.^9)^

The sensitivity and specificity of video capillaroscopy in detecting small vessel disease in diabetes can vary depending on various factors such as the specific methodology used, the expertise of the operator, and the characteristics of the study population. However, video capillaroscopy is generally considered a valuable tool for assessing microvascular changes in diabetes.^10)^ Although rare studies have evaluated the sensitivity and specificity values for video capillaroscopy in detecting small vessel disease in diabetes,^11)^ several studies have reported high sensitivity and specificity of digital imaging in detecting diabetic retinopathies.^12)^ It is important to note that these values may vary across different studies and populations. Additionally, the interpretation of video capillaroscopy images requires expertise and training. Therefore, it is recommended to consider these factors when assessing the sensitivity and specificity of video capillaroscopy in detecting small vessel disease in diabetes. The aim of the present study was to evaluate microvascular changes, including microvascular architecture, capillary distribution (morphology and density), and angiogenesis conditions in T2DM patients via video capillaroscopy.

## Materials and Methods

### Study design

We designed a retrospective case–control study with descriptive variables. The study population consisted of 256 patients suffering from T2DM referred to Loghman-Hakim Hospital, Tehran, Iran. The study population was divided into two groups. The case group consisted of patients with normal electromyography (EMG) tests and the control group consisted of patients with abnormal EMG tests. While capillaroscopic findings can be influenced by the person conducting the examination and may affect interpretation, the use of a validated report can help to create a more consistent and standardized interpretation. In diabetic patients, common capillaroscopic findings include tortuosity, bushy capillary, neoformation, bizarre capillary, microhemorrhage, capillary ectasia, and aneurysm.^13)^

### Microalbuminuria

One of the important factors in the diagnosis and management of diabetes is the evaluation of microalbuminuria levels in 24-hour urine. In this study, microalbuminuria was checked in a urine biochemistry laboratory.

### EMG–nerve conduction velocity (NCV) test

EMG and NCV are electrodiagnostic tests that measure the electrical activity of muscles and nerves. In the present study, EMG–NCV test was performed for all the patients. Based on the results of EMG–NCV test, patients were divided into two subgroups: normal EMG and abnormal EMG patients.

### Video capillaroscopy investigation

To evaluate microvascular changes in diabetic patients, video capillaroscopy was performed and related factors, including microvascular architecture, capillary distribution (morphology and density), and angiogenesis conditions, were investigated. The optical microscope that was used incorporated a video camera with ×300 magnification and a ring of green light-emitting diodes to provide high-contrast illumination of the blood vessels. The output from this video camera was fed via a (FASTCAM SA-Z type 2100K-M-64GB, Photron, San Diego, CA, USA) interfaced with a computer.

### Statistical analysis

Data were analyzed using (SPSS Inc., Chicago, IL, USA). Chi-square test, T-test, and Mann–Whitney U test were used to demonstrate differences between observed variables. Also, p-value <0.05 was considered statistically significant.

## Results

### Microalbuminuria results

A total of 256 patients enrolled in this study. Gender distribution was 56 male (22%) and 200 female (78%) patients. Mean age in patients was 58.2 (standard deviation [SD] = 12.06). The mean age was 57.73 (SD = 10.7) in males and 58.44 (SD = 11.9) in females. Comparison of age was not statistically significant between males and females, based on the Mann–Whitney test (p-value = 0.9). Microalbuminuria evaluation was performed for 117 patients. The mean levels of microalbuminuria were 207.80 mg/24 h with minimum and maximum of 4 and 4275 mg/24 h, respectively. Mean levels of microalbuminuria were higher in males in comparison to those of females (372.81 mg/24 h vs. 181.66 mg/24 h), but the difference was not significant (p-value = 0.59). The study participants were divided into the two groups (normal EMG and abnormal EMG) based on the EMG–NCV test. The results of microalbuminuria and its correlation with EMG test are shown in **Table 1**. The results indicate that the differences between microalbuminuria were not significant in patients with normal and abnormal EMG (p-value = 0.95). Also, there is no significant difference between age and EMG in the patients (p-value = 0.06).

**Table:** Table 1 Correlation of microalbuminuria and age with EMG changes.

Parameter	EMG test		p-value
	Normal (n = 36)	Abnormal (n = 81)	
Microalbuminuria (mean, mg/24 h)	324.06	142.74	0.95
Age (mean, years)	52.71	59.31	0.06

EMG: electromyography

Moreover, microalbuminuria was surveyed in the two groups, including microalbuminuria less than 300 mg in 24 h and more than 300 mg in 24 h. Subsequently, the distribution of cases with normal and abnormal EMG status was assessed for these two groups. The results showed that the distribution of subjects with microalbuminuria greater than 300 mg in 24 h is more common in the group of abnormal EMG, while those with microalbuminuria less than 300 mg in 24 h are mainly in the normal EMG group, and the difference is statistically significant (p-value = 0.037). The odds ratio showed that patients with greater microalbuminuria were at risk of abnormal EMG 2.8 times higher than those with fewer microalbuminuria (odds ratio = 2.804; 1.034–7.601).

### Comparison of microalbuminuria

#### Capillaroscopy investigations

**Figure 1:**
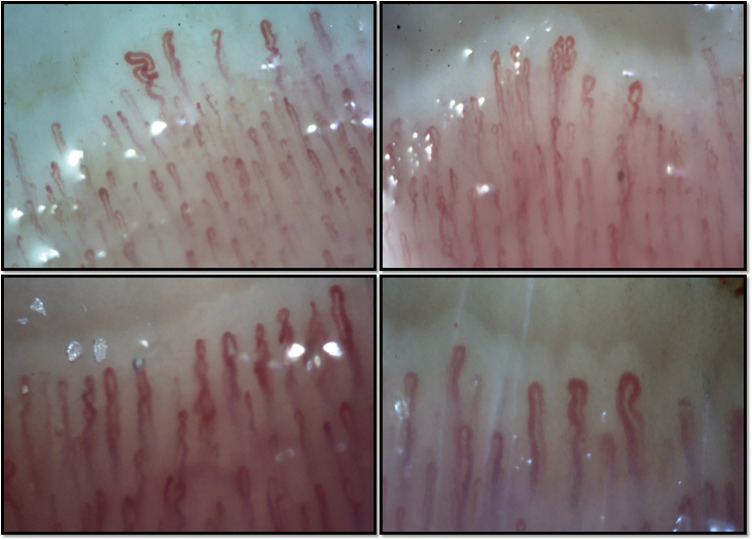
Fig. 1 Four examples of capillaroscopic changes in patients with diabetes.

Video capillaroscopy was performed to evaluate microvascular changes in diabetic patients. The frequency of different types based on video capillaroscopy is shown in **Table 2**. As the results indicate, the most common type belonged to nonspecific microangiopathy (NSMA). Moreover, 120 patients with abnormal EMG had NSMA type based on capillaroscopy. Also, the distribution of morphological abnormalities achieved in video capillaroscopy is shown in **Table 3**. These distributions show a significant difference (p-value = 0.006) between normal EMG and abnormal EMG groups. The results indicate that most people experienced angiogenesis and tortuosity simultaneously or tortuosity alone. Four examples of capillaroscopic changes in patients with diabetes are shown in **Fig. 1**.

**Table:** Table 2 Distribution of capillaroscopy results.

Results of capillaroscopy		EMG	
		Normal	Abnormal
Normal	Number	1	6
	Distributing to capillaroscopy	14.3%	85.7%
NSMA	Number	11	120
	Distributing to capillaroscopy	8.4%	91.6%
Scleroderma pattern (late)	Number	0	1
	Distributing to capillaroscopy	0.0%	100.0%
Scleroderma pattern (active)	Number	0	1
	Distributing to capillaroscopy	0.0%	100.0%
Scleroderma pattern (early)	Number	3	23
	Distributing to capillaroscopy	11.5%	88.5%
Total	Number	16	151
	Distributing to capillaroscopy	9.6%	90.4%

EMG: electromyography; NSMA: nonspecific microangiopathy

**Table:** Table 3 Distribution of morphological abnormalities.

Morphological abnormalities		EMG		Total
		Normal	Abnormal	
Angiogenesis, tortuosity	Number	1	34	35
	%	2.9	97.1	100.0
Angiogenesis, isolated microbleeding, tortuosity	Number	0	1	1
	%	0.0	100.0	100.0
Angiogenesis, avascular area (dropout), irregularly enlarged loops, tortuosity	Number	0	1	1
	%	0.0	100.0	100.0
Angiogenesis, irregularly enlarged loops, tortuosity	Number	1	0	1
	%	100.0	0.0	100.0
Angiogenesis, irregularly enlarged loops, tortuosity	Number	1	2	3
	%	33.3	66.7	100.0
Angiogenesis, isolated enlarged loops, tortuosity	Number	0	1	1
	%	0.0	100.0	100.0
Angiogenesis, isolated enlarged loops, tortuosity, microbleeding	Number	1	0	1
	%	100.0%	0.0%	100.0%
Angiogenesis, tortuosity	Number	0	1	1
	%	0.0%	100.0%	100.0%
Irregularly enlarged loops, tortuosity	Number	1	2	3
	%	33.3%	66.7%	100.0%
Isolated microbleeding, tortuosity	Number	0	1	1
	%	0.0%	100.0%	100.0%
Tortuosity	Number	2	22	24
	%	8.3%	91.7%	100.0%

EMG: electromyography

#### Microvascular architecture status

Microvascular architecture assessment showed that renal vascular structure might be altered in T2DM. Here, 165 of 256 patients were checked for microvascular architecture, and the results showed that 119 patients (72.1%) were normal and 46 patients (27.8%) had alternations in microvascular architecture. Among 119 normal patients, 108 subjects (90.7%) and among 46 abnormal patients, 42 subjects (91.3%) were in the abnormal EMG group. There was no statistically significant difference between normal and abnormal EMG groups in terms of alternation in microvascular architecture (p-value = 0.91); therefore, EMG is not a risk factor for microvascular architecture alternation in T2DM (odds ratio = 1.069; 0.323–3.546).

#### Capillary morphology status

Capillary morphology showed the morphology of tissue vessels and could be homogenous and nonhomogenous. Here, 165 of 256 patients were checked for capillary morphology and the results showed that 161 patients (97.5%) were homogenous and only 4 patients (2.5%) were non-homogenous. Of 161 normal patients with homogenous status, 146 subjects (90.6%) and of four non-homogenous patients, two subjects (50%) were in the abnormal EMG group. There was no statistically significant difference between normal and abnormal EMG groups in abnormal capillary morphology (p-value = 0.65).

#### Capillary density status

Capillary density demonstrates the density of tissue vessels and can be normal or reduced. Here, 165 of 256 patients were checked for capillary density and the results showed that 161 patients (97.5%) had normal density and only 4 patients (2.5%) had reduced capillary density. Among 161 normal patients with normal density, 146 subjects (90.6%) and among 4 non-homogenous patients, all 4 subjects (100%) were in the abnormal EMG group. There was no statistically significant difference between normal and abnormal EMG groups in abnormal capillary density (p-value = 0.52).

#### Efferent to afferent ratio

Comparison of renal efferent and afferent arteries was performed between study groups. Efferent to afferent ratio was normal or increased. Here, 165 of 256 patients were checked for efferent to afferent ratio and the results showed that 163 patients (98.7%) had a normal ratio and only two patients (1.3%) had an increased ratio. Among 163 normal patients with a normal ratio, 148 subjects (90.7%) and among two patients with an increased ratio, all two subjects (100%) were in the abnormal EMG group. There were no statistically significant differences between normal and abnormal EMG groups in the efferent to afferent ratio (p-value = 0.65). **Table 4** shows a correlation between capillaroscopy findings, and EMG and microalbuminuria status.

**Table:** Table 4 Correlation of capillaroscopic findings in correlation with EMG and microalbuminuria status.

Capillaroscopy findings	Normal urine protein and EMG	Abnormal EMG	Abnormal microalbuminuria	Both microalbuminuria and EMG abnormal
Normal	1 (5.26%)	6 (2.46%)	4 (1.62%)	3 (2.24%)
NSMA	7 (36.84%)	120 (49.18%)	109 (44.13%)	61 (45.52%)
Scleroderma (early)	3 (15.79%)	23 (9.43%)	19 (7.69%)	13 (9.7%)
Scleroderma (active)	0 (0.00%)	1 (0.41%)	3 (1.21%)	1 (0.75%)
Scleroderma (late)	0 (0.00%)	1 (0.41%)	1 (0.41%)	1 (0.75%)
Architecture abnormalities (altered)	3 (15.79%)	42 (17.21%)	41 (16.6%)	27 (20.15%)
Capillary density decrease	0 (0.00%)	4 (1.64%)	2 (0.81%)	1 (0.75%)
Enlarged and mega capillary	2 (10.53%)	6 (2.46%)	8 (3.24%)	3 (2.24%)
Microhemorrhage	1 (5.26%)	1 (0.41%)	3 (1.21%)	
Angiogenesis	2 (10.53%)	40 (16.39%)	57 (23.08)	24 (17.9%)
Total	19 (100%)	244 (100)	247 (100%)	134 (100%)

EMG: electromyography; NSMA: nonspecific microangiopathy

## Discussion

Microvascular changes are one of the most important clinical findings in diseases involving collagen vessels. These changes can lead to clinical signs and structural abnormalities.^7)^ There are various ways to measure microvascular alternations, including video capillaroscopy. It is a well-known imaging technique for diagnostic purposes in rheumatology and other metabolic diseases that characterizes the microcirculation within the body.^14^^,^^15)^ In the present study, the EMG index was used to determine neuropathic damage in diabetic patients. In terms of gender distribution, the percentage of abnormal EMGs in men is somewhat higher than that of women (97.4% in men compared to 85.7% in women), and this difference is statistically significant. However, according to the odds ratio, women are more likely than men to have a higher chance and an average of 6.16-fold (between 0.81- and 47.51-fold) for abnormal kidney involvement. In other similar studies, in addition to considering EMG, NCV is also used as a neuropathic index in diabetic patients. In Kakrani et al.,^15)^ 50 patients with diabetes were screened for neuropathic complications using the NCV index. All of these patients have T2DM at 30-years-old onset age. In this study, based on the results of the NCV test, 100% of the patients had lower extremity involvement and 48% of the patients had concomitant upper limb involvement. Overall, the results of this study have shown that distal symmetrical polyneuropathy is the most commonly diagnosed diabetic nephropathy in patients.^15)^ In the present study, 256 patients, including 56 (22%) males and 200 (78%) females, were enrolled. One of the variables associated with diabetes is a 24-hour urine test. In the present study, 24-hour microalbuminuria results showed that there is no significant relationship between the amount of this factor and the age and sex of the subjects. In similar studies, the results of 24-hour urine tests in patients with diabetes indicate that the mean 24-hour urine output in diabetics is somewhat higher than that in healthy subjects, but there is no significant correlation between uric acid and its relationship with age and sex. One of these studies performed by Zhu et al. reported no significant difference in urinary interactions between 24-hour compounds in diabetic and healthy subjects.^16)^

In the present study, patients with diabetes who were divided into two groups of normal and abnormal EMGs were examined for different modes of video capillaroscopy. The most frequent distribution of capillaroscopic findings is related to NSMA, with an incidence of 91.6% among patients with abnormal EMG, while among normal EMG patients, an incidence of 8.4% was reported. The results of this study on microangiopathy were similar to those of Iranian study performed by Rajaei et al. In a case–control study by Rajaei et al., 235 patients with T2DM who had undergone a capillaroscopy were considered for changes in capillaroscopy; the results showed that microangiopathy was present in 171 patients. Abnormal capillary abnormalities in subjects with capillary scleroderma were significantly higher than those in the control group.^17)^

Another capillaroscopy finding in our study was the frequency of scleroderma pattern in three early, active, and late stages, with the highest incidence of late scleroderma with a frequency of 23 patients (15.2%). In this study, the microvascular structure variable that identifies the renal vascular structure is investigated. The results showed that this difference was not statistically significant; this means that the chance of occurrence of microvascular changes is relatively similar and EMG cannot be a risk factor for microvascular alternations. In a similar study, Sasongko et al. evaluated microvascular changes in patients with type 1 diabetes. The results of this study indicate that early changes in the vascular position of the eye can be considered one of the main markers in predicting microvascular complications of diabetes.^18)^

## Conclusion

Collectively, our data suggest that microvascular alternations are common in T2DM and early detection of these changes could help to avoid the progress of nephropathic complications. Also, video capillaroscopy provides a promising diagnostic method in the detection of microvascular alternations in type 2 diabetes.

## Ethics Approval

All parents of infants provided written informed consents and the project was approved by the Local Committee on Health Research Ethics (Shahid Beheshti University of Medical Sciences and Health Services) with the ethics code IR.SBUMS.REC.1400.245.

## Disclosure Statement

The authors declare that they have no conflict of interests.

## Author Contributions

- SMH: Drafting of the manuscript and contribution to the conception or design of the work.
- AR, PD, AA, FF, and NE: Acquisition and interpretation of data.
- SMH, PD, and FF: Statistical analysis, acquisition, and interpretation of data.
- SMH: Revised the final manuscript for important intellectual content.
- Critical review and revision, final approval of the article, and accountability for all aspects of the work were undertaken by all authors.
